# Uterine Hemorrhage

**Published:** 1853-03

**Authors:** M. G. Parker


ART. V.—*Uterine Hemorrhage controlled by Pressure on the Aorta.* By M. G. PARKER, M. D.

MRS. M. was confined with her seventh child, December 14, 1852; nothing unusual attended the labor, which continued only about four hours. She was delivered of a large, healthy child, and immediately requested me to hurry and put her to bed, as she always wasted so bad. I proceeded to remove the placenta in about ten minutes, but before I got it entirely removed, I discovered she was losing considerable blood; I placed her in bed and applied a bandage, and the hemorrhage ceased for a short time, but in about ten minutes she called out to me, and said she was flooding to death; I went to the side of the bed and placed my finger on her pulse and it was nearly extinct. I proceeded to apply some cloths, dipped in cold water, to the abdomen, and gave some Act. Plumbi and Alum, and had some water heated, and prepared some Decoct. Ergot, but before this could be done she had fainted twice, and at this time I could feel no pulse at the wrist; I then thought of what Prof. John Evans told the class last winter, in regard to using pressure upon the abdominal aorta. I placed my fingers as firmly on the aorta as I could, and I could feel it pulsate; in about one minute the wasting in a great measure ceased; I held on about twenty minutes, and she by this time being somewhat recovered, we gave some ergot and I removed my hand in a few minutes after, but before the ergot commenced acting she was on the brink of fainting again, and said she was wasting as bad as ever. I applied my finger, so as to make a kind of fork, over the artery, and the hemorrhage ceased instanter; I began by this time to have considerable faith in the remedy, as well as the friends of the lady; I therefore kept my hand on the aorta until the ergot commenced acting, but it did not act very strongly until I removed my hand; I suppose this was owing to the circulation being almost cut off from the uterus. She complained of her right leg very much, and it was some time before it resumed its natural feeling and temperature. I am quite certain the pressure saved the lady's life.

